# Juvenile Nasopharyngeal Angiofibroma: A Case Study on the Diagnostic and Surgical Challenges in an Adolescent Male

**DOI:** 10.7759/cureus.61667

**Published:** 2024-06-04

**Authors:** Han Grezenko, Armaan M Sobhan, Muhammad Waqas, Papuna Papuashvili, Vaishvik K Patel, Rehman Khan

**Affiliations:** 1 Medicine and Surgery, Guangxi Medical University, Nanning, CHN; 2 Translational Neuroscience, Barrow Neurological Institute, Phoenix, USA; 3 Internal Medicine, St. George's University School of Medicine, Boca Raton, USA; 4 Internal Medicine, Bahawal Victoria Hospital, Bahawalpur, PAK; 5 Internal Medicine, Tbilisi State Medical University, Tbilisi, GEO; 6 Medicine, St. George’s University, West Indies, GRD; 7 Internal Medicine, Mayo Hospital, Lahore, PAK

**Keywords:** tumor management, vascular tumors, mri, ct angiography, surgical excision, diagnostic imaging, head and neck tumors, adolescent tumors, jna, juvenile nasopharyngeal angiofibroma

## Abstract

A rare and locally aggressive vascular tumor, juvenile nasopharyngeal angiofibroma (JNA) mostly affects male teenagers. This paper describes a 14-year-old male patient who presented with lethargy and recurrent nasal bleeding, which are symptoms of JNA. CT and MRI scans confirmed a vascular mass with a significant local invasion originating from the sphenopalatine foramen. After a CT angiography, which revealed the tumor’s large blood supply and helped with efficient excision, a focused surgical strategy was designed. Histopathology verified the benign nature of the tumor, and the operation was successful and the patient had a smooth recovery. This case adds to the little literature on JNA. It highlights the need for healthcare professionals to be aware of the requirement of early identification and careful presurgical preparation in managing the illness.

## Introduction

Juvenile nasopharyngeal angiofibroma (JNA), a rare, benign, but locally aggressive tumor, is mostly found in teenage males. Approximately 0.05% of all head and neck tumors are JNA, which usually appears between the ages of nine and 19, with the maximum prevalence recorded between 14 and 16 years old [1]. This tumor starts in the nasopharynx, near the superior edge of the sphenopalatine foramen. The nasal cavity, paranasal sinuses, and skull base are nearby sites that penetrate actively. Even if JNA is rare before puberty and beyond age 25, data points to a small rise in incidence rates, which are now estimated to be between 1 in 50,000 and 1 in 100,000 people [2,3].

This case report aims to improve knowledge of JNA among medical professionals by describing the clinical presentation, diagnosis process, treatment plan, and final results of a 14-year-old male diagnosed with this difficult illness. Our case aims to draw attention to the difficulties in diagnosing and treating JNA to facilitate earlier diagnosis and better treatment results for similar patients in the future.

## Case presentation

A 14-year-old male presented to the hospital with a history of intermittent nasal bleeding for the past two years, which had worsened over the last three to four months, accompanied by increasing lethargy for one month. The initial episode of nasal bleeding occurred suddenly two years prior and was transiently managed by local physicians before recurring. After several episodes, his parents sought specialized care, leading to a referral to a tertiary care facility. At presentation, the patient reported nasal discharge and mild nasal obstruction due to blood clotting. No other significant symptoms were reported, and there was no family history of similar conditions. Physical examination revealed a barely visible swelling in the right nostril, encroaching into the nasopharynx, prompting a high suspicion of nasopharyngeal angiofibroma based on the patient’s age and symptoms.

Laboratory investigations were conducted to rule out other abnormalities and support the diagnosis. Notable findings included a hemoglobin level of 9.84 g/dL, indicating mild anemia likely due to recurrent blood loss. Other parameters within the coagulation profile, hemogram, renal, and liver function tests were within normal limits, except for an elevated alkaline phosphatase level of 354 U/L. Urine analysis showed turbid yellow coloration, with all other parameters within normal ranges. These baseline tests underscored the absence of coagulopathy or significant systemic disease.

**Figure 1:**
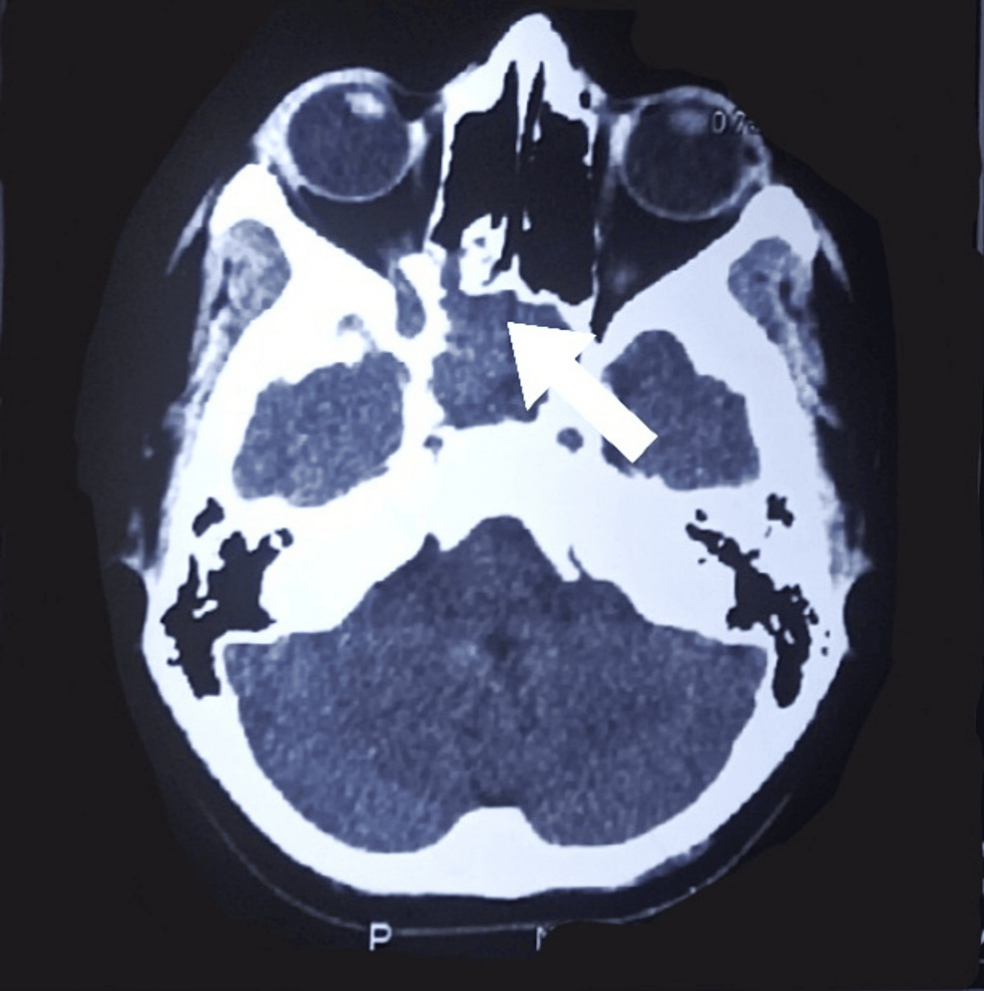
CT scan of the nose and paranasal sinuses showing the lesion extending into the right posterior nasal cavity as pointed by the arrow

The radiological assessment involved a CT scan of the nose and paranasal sinuses, which identified an avidly enhancing, ill-defined, lobulated soft tissue density lesion measuring 4 × 3.5 × 4.3 cm. This lesion originated from the right sphenopalatine foramen, causing its widening and erosion, and extended into the right posterior nasal cavity, masticator space, and bilateral sphenoid sinuses, suggesting a highly vascular JNA. The lesion can be appreciated in Figure 1. MRI with IV gadolinium further detailed the lesion’s characteristics: a soft tissue signal intensity lesion of 5.2 × 4.2 × 6.5 cm, primarily involving the nasopharynx on the right side and extending into the pterygoid palatine fossa without intracranial or oropharyngeal involvement. These imaging findings confirmed the diagnosis of JNA without signs of aggressive or metastatic disease.

**Figure 2:**
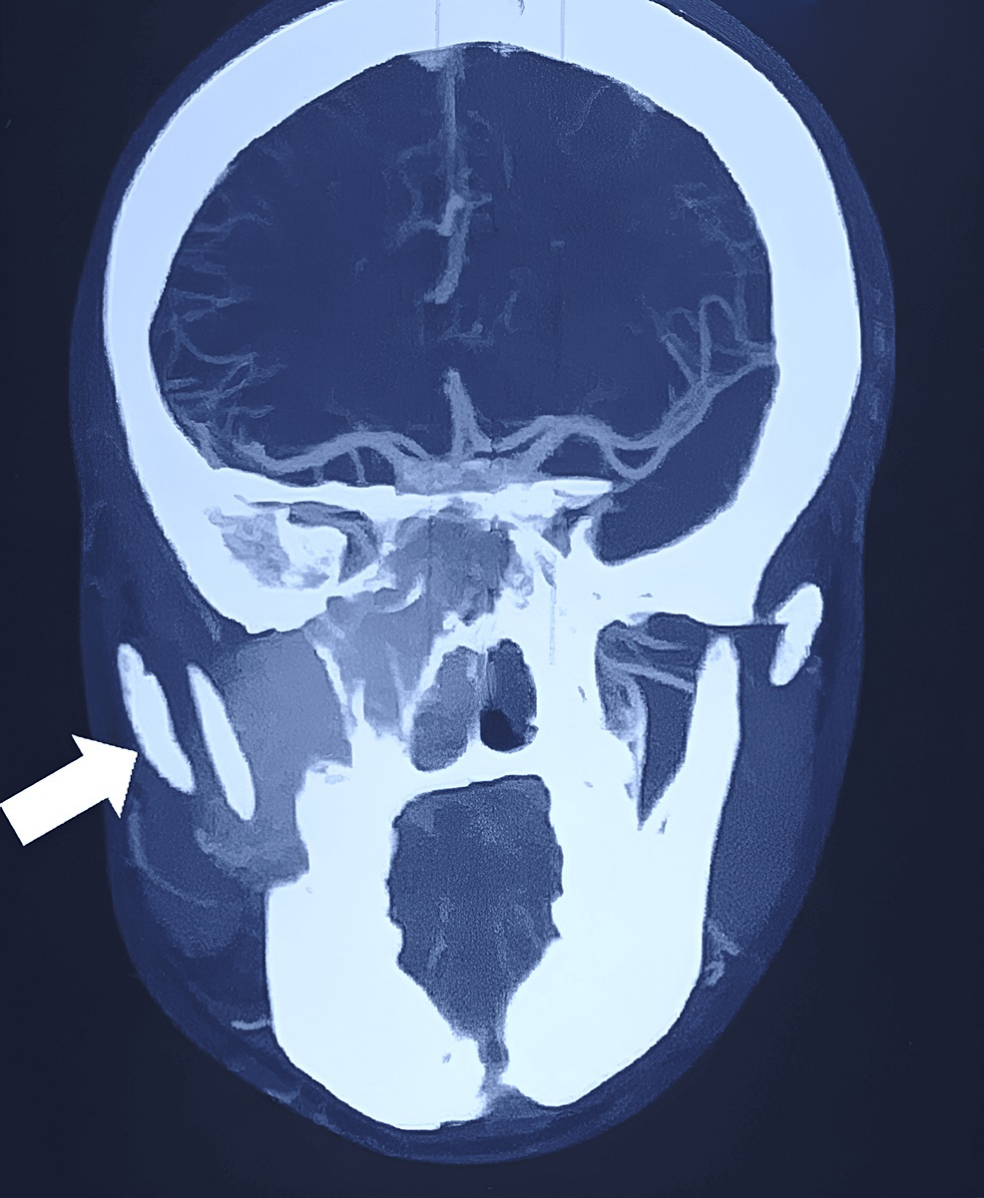
The CT angiogram revealing the sizable enhancing mass within the right side of the nasal cavity and face, as pointed by the arrow

Following the detailed imaging assessments, a CT angiogram of the carotid arteries was performed to elucidate the vascular supply of the tumor - an essential step for surgical planning. The angiogram revealed that the sizable enhancing mass within the right side of the nasal cavity and face in the maxillary region was predominantly supplied by branches of the right internal maxillary artery, as well as branches from the cavernous segments of the right internal carotid artery and the right ophthalmic artery. This information was crucial for the surgical team to plan the safest approach for tumor resection. Details of the CT angiogram are provided in Figure 2.

With the blood supply clearly defined, the medical team proceeded with the surgical excision of the angiofibroma. Prior to surgery, the patient’s low hemoglobin level, which was initially recorded at 9.8 g/dL, was addressed with a transfusion of two units of whole blood, raising his hemoglobin to 11.8 g/dL. This was a necessary intervention to ensure the patient maintained adequate oxygen-carrying capacity during the surgical procedure.

The tumor was successfully removed via the naso-oral route, and the excised tissue was sent for histopathological examination, which confirmed the benign nature of the angiofibroma, showing no malignant cells. Postoperatively, the patient was administered antibiotics for one week to prevent infection. On follow-up one week later, the patient reported significant improvement in symptoms and overall well-being, demonstrating a successful outcome of the treatment protocol. His recovery was uneventful, and he was enjoying good health, reflecting the effectiveness of the comprehensive management strategy employed for this rare and complex case.

## Discussion

One of a kind among head and neck malignancies, JNA is virtually exclusively found in teenage males and exhibits benign but locally aggressive behavior. JNA, which arises from the sphenopalatine foramen in the nasopharynx, can enter adjacent tissues, including the sinuses, skull base, and nasal cavity. Its estimated 0.05% of all head and neck tumors makes it a very uncommon disease [2]. It is consistent with the patient’s age in this case report because the usual onset age varies from nine to 19 years, peaking between 14 and 16 years. Although still uncommon, with incidences recorded between 1 in 50,000 and 1 in 100,000, evidence points to a small rise in instances, which may result from changes in reporting procedures or advances in diagnostic capabilities [2].

The clinical picture of this case emphasizes the typical JNA symptoms, including nasal discharge, nasal obstruction, and recurrent epistaxis. His low hemoglobin level of 9.84 g/dL confirms that these symptoms, together with systemic indications like tiredness and dizziness, are mostly due to anemia caused by continuous blood loss. This example emphasizes the need to consider JNA while making a differential diagnosis of teenage males’ recurrent nasal complaints.

As seen by the use of CT and MRI in this instance, diagnostic imaging is essential to assessing JNA [4]. Important for surgical planning, these modalities are also very helpful in defining the size of the tumor and its connection with surrounding structures, in addition to verifying the diagnosis. The usual JNA presentation is supported by the comprehensive imaging results showing a highly vascular lesion with distinctive extensions and erosions.

In the current literature, there is limited discussion on the outcomes of alternative treatments, such as stereotactic radiotherapy for JNA, especially in cases without intracranial extension. Given the rarity of this condition, understanding the broader spectrum of therapeutic options could provide valuable insights into nonsurgical management strategies for more complex cases [1].

JNA is characterized by robust angiogenesis and vascular proliferation within critical areas such as the posterior nasal cavity and nasopharynx. Although the precise pathophysiological mechanisms are not definitively understood, there is speculation about hormonal influences, particularly androgens during puberty and chromosomal abnormalities. These factors might explain the tumor’s aggressive behavior in adolescent males. Consequently, androgen receptor blockers like flutamide have been explored for presurgical tumor reduction, drawing parallels to their use in managing metastatic prostate cancers. This approach underscores the need for a tailored therapeutic strategy that considers the unique hormonal environment of patients [5].

The mainstay of JNA treatment, still regarded as the gold standard, is surgical resection [6]. As was done in this case, total tumor removal using the naso-oral approach is usually linked to a low risk of recurrence and a good prognosis [7]. The planned preoperative care included lowering the patient’s hemoglobin levels and cautious intraoperative management of the tumor’s vascular supply, underscoring the difficult but essential components of handling such situations. The fact that this patient’s symptoms have resolved and there are no immediate postoperative problems attests to the effectiveness of the available therapeutic approaches.

Further, the management of JNA involves a multidisciplinary approach, encompassing otolaryngologists, radiologists, and pathologists, to ensure comprehensive care. Continuous follow-up is essential due to the potential for recurrence, particularly in cases where the tumor is extensive or a complete resection is challenging.

## Conclusions

JNA, an uncommon but locally aggressive tumor that mostly affects teenage males, is described in this case report. Because JNA can cause a large local invasion and consequent blood loss, which could result in consequences including anemia, it poses serious diagnostic and treatment problems, even if it is benign. This paper highlights the need for early diagnosis and accurate imaging methods, including CT and MRI, to fully evaluate the size and vascular involvement of the tumor. The only decisive course of action is still surgical excision, which, if the tumor is removed, frequently yields a good prognosis. This case effectively handled the diagnosis to recovery, enhancing the body of knowledge on JNA and demonstrating the need for further clinical knowledge and experience in such situations.
